# Respirable controlled release polymeric colloid (RCRPC) of bosentan for the management of pulmonary hypertension: *in vitro* aerosolization, histological examination and *in vivo* pulmonary absorption

**DOI:** 10.1080/10717544.2016.1239661

**Published:** 2017-02-03

**Authors:** Lydia A. Hanna, Emad B. Basalious, Omaima N. ELGazayerly

**Affiliations:** Department of Pharmaceutics and Industrial Pharmacy, Faculty of Pharmacy, Cairo University, Cairo, Egypt

**Keywords:** Bosentan, nanoparticles, respirable controlled release polymeric colloid, intratracheal, pulmonary delivery

## Abstract

Bosentan is an endothelin receptor antagonist (ERA) prescribed for patients with pulmonary arterial hypertension (PAH). The oral delivery of bosentan possesses several drawbacks such as low bioavailability (about 50%), short duration of action, frequent administration, hepatotoxicity and systemic hypotension. The pulmonary administration would circumvent the pre-systemic metabolism thus improving the bioavailability and avoids the systemic adverse effects of oral bosentan. However, the short duration of action and the frequent administration are the major drawbacks of inhalation therapy. Thus, the aim of this work is to explore the potential of respirable controlled release polymeric colloid (RCRPC) for effective, safe and sustained pulmonary delivery of bosentan. Central composite design was adopted to study the influence of formulation and process variables on nanoparticles properties. The particle size, polydispersity index (PDI), entrapment efficiency (EE) and *in vitro* bosentan released were selected as dependent variables. The optimized RCRPC showed particle size of 420 nm, PDI of 0.39, EE of 60.5% and sustained release pattern where only 31.0% was released after 16 h. The *in vitro* nebulization of RCRPC indicated that PLGA nanoparticles could be incorporated into respirable nebulized droplets better than drug solution. Pharmacokinetics and histopathological examination were determined after intratracheal administration of the developed RCRPC to male albino rats compared to the oral bosentan suspension. Results revealed the great improvement of bioavailability (12.71 folds) and sustained vasodilation effect on the pulmonary blood vessels (more than 12 h). Bosentan-loaded RCRPC administered via the pulmonary route may therefore constitute an advance in the management of PAH.

## Introduction

Pulmonary arterial hypertension (PAH) is a life threatening disease that affects about 2–3 million people per year. It is often fatal with a mean life about three years after diagnosis (Rubin, 1997; Dingemanse & van Giersbergen, 2004; Benza et al., 2012). Symptoms comprise shortness of breath, chest pain, syncope, fatigue and peripheral edema which is more prevalent in females than in males (Saigal et al., 2013). Patients with PAH suffer from elevated levels of endothelin (ET1); a potent blood vessel constrictor, in their plasma and lung tissues (Williamson et al., 2000). ET1 reduces pulmonary arterial lumen diameter, increases pulmonary vascular resistance, decreases reactivity of the vascular bed and eventually increases pulmonary arterial pressure (Nahar et al., 2014).

Currently, endothelin receptor antagonists (ERA) are used for the treatment of PAH. Bosentan (tracleer®) was the first orally active ERA to reach the clinical investigation stage (Clozel et al., 1994; Roux et al., 1999). Bosentan is a dual ERA with high affinity of both ETA and ETB receptors. Bosentan is indicated for the treatment of pulmonary artery hypertension (PAH) by blocking the action of endothelin molecule (Ohlstein & Douglas, 1993; Vachiery & Simonneau, 2010; Granton et al., 2013) and it can prevent or reverse the associated histological change caused by elevated levels of ET1. Bosentan and its metabolites are eliminated predominantly in the liver by the cytochrome P450 isoenzymes, CYP2C9, CYP3A4 and the terminal half-life after oral administration is 5.4 h (Dingemanse & van Giersbergen, 2004). It is available in two concentrations 62.5 mg and 125 mg (for twice daily administration) and its main side effects are headache, flushing and increased liver aminotransferases. Hepatotoxicity caused by bosentan is dose-dependent in higher dosages (Krum et al., 1999; Channick et al., 2001).

Oral bosentan tablets possess several drawbacks such as low bioavailability (absolute bioavailability 50%), short duration of action necessitating frequent administration and increased levels of liver enzymes. Furthermore, since oral ERA lacks pulmonary vascular selectivity, peripheral vasodilatation and consequent systemic hypotension are common in PAH patients. Drug delivery to the lung by inhalation has been recommended due to its ability to avoid the systemic adverse effects of oral ERA. The direct application of a drug to the lungs provides targeted treatment of PAH as demonstrated for the prostacyclin analog (iloprost) which was approved by the FDA as an inhalation treatment of PAH. However, the short duration of action and the frequent administration (6 up to 12 times a day) are the major drawbacks of inhalation therapy (Kleemann et al., 2007).

Biodegradable polymeric nanoparticle colloids seem to be promising for direct delivery of nanoencapsulated therapeutics to the lung to produce sustained localized pulmonary arterial vasodilation. PAH is a disease of terminal pulmonary vasculature. Thus, a delivery system that accumulates in pulmonary bronchioles and releases drug in a controlled fashion could reduce the shortcomings of current oral PAH medications (Beck-Broichsitter et al., 2012a; Nahar et al., 2014). Nanoparticles composed of biodegradable poly(lactic-co-glycolic acid), PLGA, could escape macrophage uptake (Nahar et al., 2014), exhibit minimum lung tissue damage (Hara et al., 2008), ability to be transferred into an aerosol, stability against forces generated during aerosolization, biocompatibility, targeting of specific sites in the lung, release of the drug in a sustained manner and degradation within an acceptable period of time (Beck-Broichsitter et al., 2009, 2012a,b).

The optimum size of inhaled particles for deep lung deposition is <5 μm (Sung et al., 2007). However, particles having this relatively large size are susceptible to macrophage uptake and rapid clearance from the lung with a consequent short duration of action. It is reported that particles within a size range of <1 μm can escape macrophage uptake (Nahar et al., 2014). Thus, the size of the inhaled systems needs to be not only relatively large to be respirable in the deep lung tissues but also to be small to be retained in the lung and avoid rapid clearance. Nebulization of colloidal dispersion of PLGA nanoparticles could provide the small particles (<500 nm), necessary to achieve the prolonged therapeutic effect, incorporated in respirable droplets (<5 μm) of the dispersion to be easily deposited in the deep lung tissues. To the best of our knowledge, no attempt has been reported to investigate the capability of the polymeric colloid to form respirable droplets upon nebulization with jet-air nebulizer. The respirable droplets act as carriers for bosentan loaded PLGA nanoparticles which release the drug in a sustained manner to achieve the required prolonged pulmonary arterial vasodilation.

The aim of this research work is to explore the potential of respirable controlled release polymeric colloid (RCRPC) for effective, safe and sustained pulmonary delivery of bosentan. The effects of formulation and process variables on the entrapment efficiency (EE), particle size and zeta potential (ZP) as well as the drug release was evaluated using central composite design. The capability of RCRPC to form respirable nebulized droplets was tested in a twin stage impinger (TSI) compared to drug solution. Bosentan pharmacokinetics was determined after intratracheal administration of the developed bosentan-loaded RCRPC to male albino rats compared to oral bosentan suspension. Furthermore, histopathological examination of the rat lung tissues was investigated after oral and intratracheal administration of bosentan compared to control rat lung tissue.

## Materials and methods

### Materials

Poly(lactic-co-glycolic acid) polymer (50:50) (PURASORB® PDLG 5002A, inherent viscosity of 0.2 dL/g) was generously donated from PURAC (Gorinchem, Netherlands). Bosentan was supplied by Parabolic (Chandigarh, India). Polyvinyl alcohol (PVA) (98–99% hydrolyzed with Low Mwt.) was purchased from Alfa Aesar (Karlsruhe, Germany). Sodium lauryl sulfate (SLS) was obtained from Oxford (Mumbai, India). Dichloromethane (DCM), sodium dihydrogen phosphate, sodium hydroxide pellets and methanol were obtained from Scharlau (Barcelona, Spain). Pure acetone was purchased from El Nasr pharmaceutical Chemicals Co. (Cairo, Egypt). All solvents used were HPLC grade.

### Preliminary study for selecting the proper solvent mixture for the preparation of bosentan RCRPC

RCRPC was prepared by spontaneous emulsification solvent diffusion (SESD) method (Niwa et al., 1993; Niwa et al., 1994; Kawashima et al., 1998; Galindo-Rodriguez et al., 2004; Ganachaud & Katz, 2005; Reis et al., 2006; Esmaeili et al., 2007). Different solvent mixtures were tested to select the appropriate one for nanoparticle formation, namely; DCM/methanol (50/50) or DCM/acetone (50/50) (Kawashima et al., 1998; Soppimath et al., 2001; Song et al., 2008). The amount of PLGA and bosentan used was 300 mg and 50 mg, respectively. The concentration of PVA in the aqueous phase was 3% w/v. Selection of the most optimum solvent mixture was based on the proper emulsification and the morphology of the formed nanoparticles when examined under Leica image analyzer (Model Q 5501W) equipped with Leica DMLB microscope (Cambridge, England), connected to a camera (Model TK-C 1380 JVC, Victor Company, Yokohama, Japan).

### Preparation of RCRPC of bosentan

RCRPC was prepared using SESD method. PLGA was dissolved in 7.5 mL DCM and 50 mg bosentan was dissolved in 7.5 mL methanol. The drug solution was added to PLGA solution in DCM to form the oily phase. The organic phase was poured at once on 30 mL aqueous solution containing specified amount of PVA. The whole system was then homogenized for 10 min at previously defined rate using high speed homogenizer (Ultra-Turrax® T25 digital) (IKA®-Labortechnik, Staufen, Germany). The organic solvent was evaporated while being stirred gently at room temperature for 12 h under agitation with a magnetic stirrer (Stuart, Staffordshire, UK). The formed nanoparticles were collected using cooling ultracentrifuge (HERMIL, Wehingen, Germany) at 15 000 rpm for 1 h at 4 °C. The separated nanoparticles were washed once with 1% SLS in phosphate buffer (pH 7.4) and collected again with centrifugation at 15 000 rpm for 1 h at 4 °C. The optimized RCRPC system was frozen at −20 °C in presence of 5% w/v mannitol as a cryoprotectant. Then, samples were lyophilized at −45 °C and pressure of 7 × 10^−2^ mbar for 24 h (Novalyphe-NL 500; Savant Instruments Corp., Holbrook, NY).

### Formulation optimization of RCRPC using central composite experimental design

Central composite experimental design was employed to evaluate the individual and combined effects of the formulation and process variables using the Design-Expert® 7 software. In this design, two formulation and one process factors were evaluated. The amount of PLGA polymer (*X*_1_), the concentration of PVA in the aqueous phase (*X*_2_) and the stirring rate (*X*_3_) were selected as independent variables. The chosen dependent variables were the particle size, PS (*Y*_1_); polydispersity index, PDI (*Y*_2_); encapsulation efficiency, EE (*Y*_3_) and *Y*_4_, *Y*_5_ and *Y*_6_ represent the amount of bosentan *in vitro* released after 0.5, 8 and 16 h, respectively. Each numeric factor is varied over five levels as follows; axial points (+alpha and − alpha), factorial points (+1 and −1) and center point. Table 1 depicts the composition of the prepared RCRPC of bosentan.

**Table 1.** Composition and characterization of the prepared bosentan RCRPC based on central composite design.

										
Formulation #	PVA conc. (*X*_2_) (%)	PVA conc. (*X*_2_) (%)	Stirring rate (*X*_3_) (rpm)	Particle size (nm) *Y*_1_	PDI *Y*_2_	EE (%) *Y*_3_	Q0.5 (%) *Y*_4_	Q8 (%) *Y*_5_	Q16 (%) *Y*_6_	Zeta potential (mV)
RCRPC 1	400.00	5.00	7000.00	1225.0 ± 3.53	0.514 ± 0.01	80.49 ± 0.01	0.50	13.86	24.45	−12.20 ± 0.21
RCRPC 2	300.00	2.31	8500.00	394.2 ± 9.82	0.404 ± 0.01	35.36 ± 0.09	0.75	29.30	45.55	−25.30 ± 0.28
RCRPC 3	200.00	5.00	7000.00	913.8 ± 4.45	0.580 ± 0.01	63.12 ± 0.26	0.94	10.41	15.77	−13.30 ± 0.14
RCRPC 4	300.00	4.00	8500.00	552.3 ± 1.48	0.481 ± 0.01	47.82 ± 1.67	1.10	21.86	38.56	−18.70 ± 0.70
RCRPC 5	300.00	4.00	8500.00	373.7 ± 4.45	0.360 ± 0.01	32.18 ± 1.192	1.02	23.00	25.19	−20.80 ± 0.28
RCRPC 6	200.00	5.00	10000.00	532.6 ± 5.30	0.685 ± 0.01	82.19 ± 0.63	0.80	15.26	24.42	−16.70 ± 0.14
RCRPC 7	300.00	4.00	8500.00	438.9 ± 6.15	0.400 ± 0.01	55.32 ± 0.79	1.30	29.80	42.00	−19.10 ± 0.14
RCRPC 8	300.00	4.00	8500.00	412.3 ± 1.55	0.404 ± 0.01	55.87 ± 0.40	1.57	22.96	35.67	−18.60 ± 0.49
RCRPC 9	200.00	3.00	7000.00	1130.0 ± 1.41	0.560 ± 0.01	29.75 ± 0.18	1.10	27.10	42.20	−13.90 ± 0.14
RCRPC 10	200.00	3.00	10000.00	554.7 ± 0.84	0.530 ± 0.01	30.74 ± 0.32	0.77	10.54	19.58	−16.90 ± 0.07
RCRPC 11	400.00	5.00	10000.00	497.7 ± 0.98	0.455 ± 0.01	76.50 ± 0.70	0.52	13.74	20.03	−14.90 ± 0.42
RCRPC 12	400.00	3.00	7000.00	1259.0 ± 5.65	0.541 ± 0.01	33.66 ± 0.16	1.09	14.86	27.34	−14.80 ± 0.14
RCRPC 13	468.18	4.00	8500.00	442.6 ± 5.37	0.391 ± 0.01	43.00 ± 0.14	1.10	13.00	28.00	−22.90 ± 0.49
RCRPC 14	300.00	4.00	8500.00	508.0 ± 0.21	0.504 ± 0.02	49.00 ± 0.77	1.70	23.30	39.70	−18.90 ± 0.21
RCRPC 15	400.00	3.00	10000.00	519.7 ± 0.42	0.465 ± 0.01	60.10 ± 0.14	1.40	15.80	25.20	−18.50 ± 0.21
RCRPC 16	300.00	5.68	8500.00	491.6 ± 2.54	0.363 ± 0.01	56.52 ± 0.25	0.50	17.17	27.03	−14.60 ± 0.21
RCRPC 17	131.82	4.00	8500.00	518.4 ± 1.48	0.560 ± 0.02	55.74 ± 1.09	1.18	14.20	19.42	−16.30 ± 0.21
RCRPC 18	300.00	4.00	11022.60	432.9 ± 3.25	0.373 ± 0.021	80.24 ± 0.25	1.70	25.50	41.50	−15.40 ± 0.07
RCRPC 19	300.00	4.00	8500.00	312.2 ± 7.07	0.266 ± 0.01	38.00 ± 0.070	1.20	20.00	35.00	−23.60 ± 0.28
RCRPC 20	300.00	4.00	5977.31	1350.0 ± 0.70	0.600 ± 0.02	91.12 ± 0.72	1.30	29.20	30.60	−19.60 ± 0.35

*X*_1_, *X*_2_ and *X*_3_ are the independent variables.

*Y*_1_, *Y*_2_, *Y*_3_, *Y*_4_, *Y*_5_ and *Y*_6_ are the dependent variables (responses).

### Characterization of the prepared bosentan RCRPC

#### Encapsulation efficiency (EE)

A weighed amount of the washed residue of RCRPC was dissolved in DMSO. Filtration was performed using a 0.45 mm filter. A 2 μL aliquot of the filtrate was injected in triplicate into HPLC column (Zorbax SB 250 mm, 4.6 × 5 μm). Chromatography was performed using a Waters Alliance system with a UV detector at 280 nm. The column temperature was set at 40 °C and the flow rate was 1 mL/min. The mobile phase was composed of 10% acetonitrile, 50% methanol and 40% 0.05 M phosphate buffer, pH 7.5. The drug EE was calculated as the mass ratio of the amount of drug determined to the theoretical amount in RCRPC (Khowessah et al., 2009). Determinations were done in triplicates for five independent samples of each formula and the average values ± SD were calculated.

#### Determination of PS, PDI and ZP of bosentan RCRPC

Size distribution of bosentan-loaded RCRPC was determined by the dynamic light scattering method using a Malvern Mastersizer (DLS, Zetasizer Nano ZS, Malvern Instruments, Malvern, UK). The samples were diluted with distilled filtered water before measurement until being translucent. PDI was measured to assess the particle size distribution. Finally, ZP of the diluted samples was analyzed for evaluation of their physical stability. Three samples for each formula were used for size determination and the average values ± SD were calculated.

#### In vitro release study of bosentan from the prepared RCRPC

*In vitro* release study of bosentan from the prepared RCRPC was carried out at 37 °C ± 0.5 °C by a dialysis tubing cellulose (Zhang et al., 2001; Das et al., 2011; Kumbhar & Pokharkar, 2013) with a molecular weight cut off (MWCO) (12 000–14 000 Da) (Sigma, St. Louis, MO). Briefly, a specified amount of the washed residue of RCRPC equivalent to 10 mg bosentan was dispersed in 5 mL normal saline. The dispersion were placed in the dialysis bag and tied at both ends. The dialysis bag was placed in 250 mL of the release medium (1% SLS in phosphate buffer pH 7.4) and shaken in a thermostatically controlled shaker (Memmert, Büchenbach, Germany) at 100 rpm (Hu et al., 2004; Song et al., 2008). At predetermined time intervals (0.5, 1, 1.5, 2, 3, 4, 6, 8, 12, 16 and 24 h); 1 mL of the release medium was withdrawn and replaced with equal volume of fresh release medium. All samples were analyzed for drug content using the validated HPLC previously mentioned. All experiments were run in duplicates.

#### Kinetic analysis of bosentan release data

The mean *in vitro* release data of bosentan were fitted to different kinetic models (zero order, Higuchi, and Korsmeyer–Peppas) to evaluate the kinetics of drug release from the prepared bosentan loaded nanoparticles. The large value of the coefficient of determination (*R*^2^) indicated a superiority of the dissolution profile fitting to mathematical equations.

#### Morphological examination of bosentan-loaded RCRPC

The photomicroscopic examination of the optimized RCRPC was performed by a microscope using Leica image analyzer. A drop of the optimized RCRPC was placed on microscopic slide then examined and photographed for morphological evaluation.

The morphology of RCRPC was also examined using transmission electron microscopy (TEM) operating at 80 kV (model JEM-1230, Jeol, Tokyo, Japan). One drop of the optimized RCRPC was deposited on the surface of a carbon coated copper grid, then allowed to dry at room temperature for 10 min for investigation by TEM.

#### Differential scanning calorimetry (DSC)

The thermal analysis of pure bosentan, PLGA, mannitol, physical mixture (PM) and lyophilized optimized formulation was determined using Shimadzu differential scanning calorimeter (DSC-50, Kyoto, Japan). Approximately, 4 mg of each sample was heated in aluminum pans in a temperature range of 25–250 °C at a heating rate of 10 °C/min under inert nitrogen flow (25 mL/min).

#### Powder X-ray diffraction (XRD)

Diffraction patterns of bosentan, PLGA, mannitol, PM and lyophilized optimized formulation were determined in a Scintag X-ray diffractometer (Coyote, CA) using Cu Ka radiation with a nickel filter, a voltage of 45 kV, and a current of 40 mA.

#### In vitro aerosol delivery of optimized RCRPC using a TSI and a jet-air nebulizer

The nebulization of RCRPC dispersion, bosentan solution in phosphate buffer containing 1% SLS as well as bosentan suspension in normal saline were carried out using a jet-air nebulizer (Medel® easy, Aerosol Therapeutic System, Parma, Italy). The aerodynamic behavior of the generated aerosols was evaluated using the twin-stage impinger (TSI) (Copley Instruments, Nottingham, UK) following the stated procedure in British Pharmacopoeia (2014). Three collection stages were considered (Liu et al., 2008). The upper stages (stages 0 and 1) represent the upper airways, and the lower stage (stage 2) represents lower respiratory airways. A flow rate was adjusted using a flow meter attached to the vacuum pump at 60 L/min (Model HCP5, Copley Instruments, Nottingham, UK). This flow rate provides a cutoff aerodynamic diameter of 6.4 μm between the two stages of the impinger. Each liquid formulation (2 mL containing about 4 mg bosentan) was filled into the nebulizer cup. A 7 and 30 mL of methanol/propylene glycol mixture (60:40) were introduced into the upper and lower impingement chambers, respectively. The nebulizer was operated until aerosol generation completely ceased. The liquid in the lower and upper chambers was analyzed for drug content using the validated HPLC method previously mentioned. The nebulizer cup was washed with acetonitrile and the washing was assayed for drug content which represented “the unemitted fraction”.

The nebulization efficiency (NE) was determined as the ratio of the amount of drug collected from the two impinger stages and the amount of the drug in the formulation loaded into the nebulizer cup. The fine particle fraction (FPF) and the alveolar respirable fraction (ARF) were determined using the following equations (Abd-Elbary et al., 2008; Liu et al., 2008; Nasr et al., 2012):

(1) $$\mathrm{Fine}\;\mathrm{particle}\;\mathrm{fraction}\;(\mathrm{FPF})=\frac{\mathrm{Amount}\;\mathrm{of}\;\mathrm{bosentan}\;\mathrm{deposited}\;\mathrm{in}\;\mathrm{the}\;\mathrm{lower}\;\mathrm{impinge}\;\mathrm{chamber}}{\mathrm{Amount}\;\mathrm{of}\;\mathrm{bosentan}\;\mathrm{in}\;\mathrm{RCRPC}\;\mathrm{loaded}\;\mathrm{in}\;\mathrm{the}\;\mathrm{nebulizer}\;\mathrm{cup}}$$

(2) $$\mathrm{The}\;\mathrm{alveolar}\;\mathrm{respirable}\;\mathrm{fraction}\left(\mathrm{ARF}\right)=\;\frac{\mathrm{Fine}\;\mathrm{particle}\;\mathrm{fraction}\;(\mathrm{FPF})}{\mathrm{Nebulization}\;\mathrm{efficiency}\;(\mathrm{NE})}$$

The ARF corresponds to the fraction of the emitted drug desirable for alveolar deposition. NE, ARF and FPF were employed as parameters to evaluate the aerodynamic behavior of RCRPC of bosentan.

#### In vivo pulmonary absorption of bosentan RCRPC

*Study design*. The *in vivo* study was carried out to determine the pharmacokinetics of bosentan in the plasma after intratracheal administration of RCRPC compared to oral administration of bosentan suspension. The protocol of this study was reviewed and approved by the Research Ethics Committee (REC) at Faculty of Pharmacy, Cairo University (Cairo, Egypt). The study was done using Wister male Albino rats (270–300 g). Before initiation of the experiment, the animals were fasted for 10 h with free access to water.

*Plasma PK study*. Sixteen Wister male albino rats were divided into two groups, each comprising eight rats, before the experiment. The animals were anesthetized with I.P. injection of ethyl carbamate (0.175 mg/100 g of rat weight) (Kodani et al., 2000; Moldestad et al., 2009). Group 1 received 0.5 mL of oral bosentan suspension in 1% HPMC solution (equivalent to 1 mg bosentan). Group 2 received RCRPC of bosentan by intratracheal injection. With the animal secured on its back, the trachea was exposed and then an incision was made between the sixth and fifth trachea rings (Enna & Schanker, 1972; Okumura et al., 1992; Zhang et al., 2001,2011; Hara et al., 2008). 0.1 mL of RCRPC with 0.1 mL air was injected directly into trachea through a microsyringe with PE tubing (Intramedic® PE-50/36”) inserted in the incision. The procedure for injection of RCRPC with air was repeated five successive times for intra-tracheal injection of 0.5 mL RCRPC (equivalent of 1 mg bosentan). The rats were kept at an angle of 90° to horizontal for 30 s. after the intra-tracheal administration. Blood samples (∼1 mL) were obtained from the carotid artery using (three valve cannula) by a well-trained practitioner. The samples were collected into heparinized tubes prior dosing and at 0.5, 1, 2, 3, 4, 6, 8, 10 and 12 h. after administration. Plasma was harvested immediately by 20 min of centrifugation at 7000 rpm and stored at −80 °C until analysis. Rat sacrifice was performed using over dose of anesthesia and lung was dissected and was used for histopathological examination.

*Sample preparations*. A volume of 10 μL of IS (Torsemide 4 μg/mL) was added to 100 μL rat plasma, then the samples were vortexed for 30 s. Precipitation was applied by adding 200 μL acetonitrile, followed by vortex for 1 min, then samples were centrifuged at 3000 rpm for 10 min. A volume of 5 μL of the supernatant was injected into the UPLC–MS/MS system.

*Chromatographic conditions*. Plasma samples were analyzed for bosentan adopting a sensitive and accurate LC–MS/MS method, developed and validated before the study using UPLC–MS/MS system. Quantitative analysis was performed on a Waters Acquity UPLC H-Class-Xevo TQD system (MA, USA) interfaced with a Waters Quattro Premier XE triple quadrupole mass spectrometer and equipped with electrospray ionization operated in the positive ionization mode. Chromatographic separation of analytes was carried out on ACQuity UPLC CSH C18 (50 × 2.1 mm, 1.7 μm) column. The isocratic mobile phase composed of acetonitrile −0.1% formic acid (70:30 (v/v)) and was delivered at a flow rate of 0.35 mL/min. The column was maintained at 40 °C and the pressure of the system was 6500 psi. The source dependent parameters maintained for both the analytes and internal standards (ISs) were: cone gas flow, 50 L/h; desolvation gas flow, 800 L/h; capillary voltage, 3.5 kV, source temperature, 120 °C; desolvation temperature, 350 °C. The optimum values for compound dependent parameters like cone voltage and collision energy were set at 55 V and 34 eV for NM and 30 V and 18 eV for IS, respectively. The mass transition ion pair, performed in the multiple-reaction monitoring (MRM) mode, of *m*/*z* 552.207 → 202.10 was followed for bosentan and *m*/*z* 349.14 > 264.10 for IS. Mass Lynx software version 4.1 was used to control all parameters of UPLC and MS. The lower and upper limits of quantification of bosentan in plasma samples were 1–2500 ng/mL.

*Pharmacokinetic and statistical analyses*. Plasma concentration–time data of bosentan was analyzed by noncompartmental pharmacokinetic models using Phoenix® WinNonlin® 6.4 (Certara, L.P., St. Louis, MO). Concentration–time profiles were plotted and the peak concentration *C*_max_ (ng/mL) and the necessary time *T*_max_ (h) to attain *C*_max_ were obtained directly from it. The area under the drug concentration–time curve (AUC) was calculated by the trapezoidal method. The terminal elimination rate constant was calculated by linear regression of the terminal portion of the natural logarithm of the concentration and the elimination half-life was calculated. The pharmacokinetic data obtained from different treatments were analyzed for statistical significance by one-way analysis of variance (ANOVA). The non-parametric test (Kruskal–Wallis test) was used to compare the *t*_max_ for test and reference.

#### Histological examination of rat lung tissue

In order to detect the histological changes that may occur to the lungs due to administration of RCRPC, histological staining of the lungs was performed. Autopsy lung samples were taken 12 h post dosing from the two different groups of rats enrolled into the *in vivo* pulmonary absorption study compared to third group that was administered phosphate buffered saline intratracheally as a negative control. Autopsy samples were taken from the lung of rats and fixed in 10% formal saline for 24 h. Washing was done in tap water then serial dilutions of alcohol (methyl, ethyl and absolute ethyl) were used for dehydration. Specimens were cleared in xylene and embedded in paraffin at 56 °C in hot air oven for 24 h. Paraffin bees wax tissue blocks were prepared for sectioning at 4 μm thickness by slidge microtome. The obtained tissue sections were collected on glass slides, deparaffinized, stained by hematoxylin and eosin stain for routine examination through the light electric microscope (Nasr et al., 2013) (Axiostar plus, Zeiss, New York, NY).

### Statistical analysis

The data obtained from different formulations were analyzed for statistical significance by one-way ANOVA adopting SPSS statistics program (version 19, SPSS Inc., Chicago, IL) followed by post hoc multiple comparisons. Differences were considered to be significant at *p* < 0.05.

## Results and discussion

### Selection of the proper solvent mixture for SESD

Bosentan RCRPC was prepared using SESD method (Niwa et al., 1993; Niwa et al., 1994; Kawashima et al., 1998; Galindo-Rodriguez et al., 2004; Ganachaud & Katz, 2005; Reis et al., 2006; Esmaeili et al., 2007). The solvent mixture used in this method should be composed of water miscible solvent such as methanol or acetone together with a water immiscible organic solvent (DCM is most commonly used) as the organic phase. The proper selection of the solvent mixture is very crucial step for the successful development of nanoparticle colloid having optimum particle size and maximum encapsulation efficiency of the drug. Homogenization of the organic solvent mixture into the aqueous phase containing PVA as stabilizer results in an interfacial turbulence between the two phases due to the spontaneous diffusion of water miscible solvent into water leading to the formation of small particles (Alex & Bodmeier, 1990; Soppimath et al., 2001; Khowessah et al., 2009). Two solvent mixtures (DCM/acetone or DCM/methanol in ratio 1:1 by volume) were tested for their efficiency in nanoparticle formation by SESD. The morphology of the prepared nanoparticle colloid was examined by image analyzer microscope. The nanoparticles prepared by DCM/methanol were discrete, non-agglomerated and spherical in shape. However, DCM/acetone showed no nanoparticle formation and the drug was precipitated as crystals. The poor capability for nanoparticle formation using DCM/acetone solvent mixture could be explained based on the drug solubility in acetone. Bosentan monohydrate is freely soluble in acetone and DCM, soluble in ethanol and ethyl acetate, slightly soluble in methanol and isopropanol (European Public Assessment Report of Tracleer, 2004). Once organic solvent mixture was added to the aqueous phase, acetone rapidly diffused into the aqueous phase withdrawing bosentan from the polymeric matrix. The rapid depletion of the drug from the polymeric matrix hindered the formation of nanoparticles and resulted into the precipitation of bosentan crystals in the external phase. On the other hand, the displacement of methanol into the external aqueous phase resulted in the proper formation of nanoparticles due to the relatively low solubility of bosentan in methanol. Thus, DCM/methanol was used for the further preparation of RCRPC of bosentan.

### Formulation optimization of bosentan-loaded RCRPC using central composite experimental design

In order to obtain the optimal RCRPC, central composite experimental design was applied in this study. The amount of PLGA polymer (*X*_1_) and the concentration of PVA in the aqueous phase (*X*_2_) were chosen as formulation variables and the stirring rate (*X*_3_) was chosen as the only process variable. The particle size (*Y*_1_), PDI (*Y*_2_) encapsulation efficiency (*Y*_3_), and the percentage of bosentan *in vitro* released after 0.5, 8 and 16 h (*Y*_4_, *Y*_5_ and *Y*_6_, respectively) were used as the responses. The responses of these formulations are summarized in Table 1. The independent and response variables were related using polynomial equation with statistical analysis through Design-Expert® software. The approximation of all response values (*Y*_1_, *Y*_2_, *Y*_3_, *Y*_4_, *Y*_5_ and *Y*_6_) based on quadratic model was the most suitable method because its PRESS was smallest. The values of the coefficients *X*_1_, *X*_2_ and *X*_3_ are related to the effect of these variables on the responses (data are not shown). A positive sign of coefficient indicates a synergistic effect while a negative term indicates an antagonistic effect upon the response (Fouad et al., 2013). The larger coefficient values mean that the independent variable has more potent influence on the response.

### Investigation of formulation and process variables on the properties of bosentan-loaded RCRPC

#### Effect on particle size (Y_1_), PDI (Y_2_) and ZP of bosentan polymeric nanoparticles

As shown in Table 1, the particle size of the different formulations of polymeric nanoparticles varied between 312 nm and 1350 nm. It can be inferred that formulation factors did not have a profound effect on the particle size. The process factor (stirring rate *X*_3_) had a significant reductive effect on the particle size of nanoparticle (*p* < 0.0001). Increasing the stirring rate provided the necessary shearing energy required to break down the large particles into smaller ones (Yang et al., 2001; Rahman et al., 2010). ANOVA of the effect of variables on PDI of polymeric nanoparticle colloid (*Y*_2_) showed that only the formulation variable (amount of PLGA, *X*_1_) had a significant effect on this response (*p* < 0.05). The lower PDI corresponds to a population of nanoparticles with a more homogeneous particle size distribution (Galindo-Rodriguez et al., 2004). The lowest PDI values could be obtained at the highest values of PLGA and stirring rate. Zeta potential of RCRPC was in the range of (−12 to −28.5) mV. The charge of the particles could influence the stability of the preparation. Extremely positive or negative ZP values result in larger repulsive forces (Dillen et al., 2004). The negative value corresponds to the terminal free carboxylic end group of the used PLGA. Results of ZP propose that the aggregation of the prepared nanoparticles may be prevented by the negative charge, which further enhances the physical stability in addition to the steric stabilization of the used PVA.

#### Effect on EE of bosentan in the polymeric nanoparticles (Y_3_)

As shown in Table 1, EE (%) of the prepared RCRPC ranged between 29.7% and 91.12%. ANOVA of the effect of formulation and process variables on EE (%) of the polymeric nanoparticles (*Y*_3_) showed that only the formulation variable (PVA concentration, *X*_2_) exhibited a significant effect on this response (*p* < 0.0004). The EE increased significantly with the increase of PVA concentration. As the concentration of PVA increases, the number of PVA molecules adsorbed onto the surface of nanoparticles will be increased resulting in formation of thick film via inter- or intra-molecular bonds. This thick film together with the high viscosity of concentrated PVA aqueous phase would possibly delay the diffusion of the drug into the external aqueous layer, thus increasing the encapsulation efficiency (Li et al., 2000; Hong et al., 2001; Lewandowska et al., 2001; Lyoo et al., 2003; Song et al., 2008).

#### Effect on in vitro release of bosentan from nanoparticles of RCRPC

A sustained release pattern is a key factor in the development of colloidal systems used in the field of pulmonary delivery. The process of drug release from biodegradable nanoparticles is governed by diffusion and biodegradation of the polymer matrix (Beck-Broichsitter et al., 2012b). The *in vitro* release study for the biodegradable polymeric nanoparticles gives only an indication about the diffusion pattern of the drug from the polymeric matrix but does not predict the release rate due to the biodegradation of the polymeric matrix of the nanoparticles. The release profiles of bosentan from all RCRPC systems were characterized by lack of burst release where the maximum amount of bosentan released after 0.5 h was 1.7%. ANOVA of the effect of formulation and process variables on Q0.5 (%) of the drug from the polymeric nanoparticles (*Y*_4_) showed that only the formulation variable (PVA concentration, *X*_2_) had a remarkable effect on this response. The coefficient of *X*_1_*X*_2_ for both responses was largest, showing the negative effect of combination of amount of PLGA and PVA concentration on the amount released after 0.5 h from the polymeric nanoparticles. Both PVA and PLGA form a barrier against the initial diffusion of the drug from the nanoparticle matrix resulting in reduction of drug release.

Regarding drug release after 8 and 16 h, ANOVA results showed that there was no significant formulation and process variable effects on Q8 and Q16 (%) of the drug from the polymeric nanoparticles (*Y*_5_ and *Y*_6_, respectively).

The aim of the optimization of pharmaceutical formulations is generally to determine the levels of the variable from which a robust product with high quality characteristics may be produced (Basalious et al., 2010,2011). Some of the measured responses have to be minimized. In this case, these responses comprise the particle size (<500 nm), PDI (<0.5) and Q0.5 (<2%). The formulation of homogenous RCRPC of bosentan with small particle size and prolonged release profile lacking burst release of the drug allows effective pulmonary delivery of the developed nanoparticle colloidal system. Some other responses, such as the EE should be maximized (>60%) for controlled release delivery of bosentan from RCRPC. There is no proposed design space was formed if the stirring rate was below 9000 rpm. The control strategy of the optimized RCRPC was determined to ensure process performance and product quality. The control space (or normal operating ranges) is defined as the upper and/or lower limits for the formulation and process variables between which the parameters are routinely controlled during production in order to assure reproducibility. The control space should be within the design space. If the control space is much smaller than the design space, the process is then considered robust. Increasing the stirring rate above 9000 rpm remarkably increases the design space. In this case, the optimum operating ranges of the formulation variables (PVP concentration and the amount of PLGA) for robust development of RCRPC are 3.9–4.5% and 250–350 mg, respectively (figure not shown). A RCRPC of bosentan satisfying these criteria was prepared and evaluated. An optimum response was found with *Y*_1_, *Y*_2_, and *Y*_3_, *Y*_4_, *Y*_5_ and *Y*_6_ of 420 nm, 0.39, 60.5%, 1.2%, 19.3% and 31.0% at *X*_1_, *X*_2_ and *X*_3_ values of 334.74 mg, 4.1% and 9900 rpm, respectively.

### Morphological examination of the optimized bosentan-loaded RCRPC

**Figure 1.**
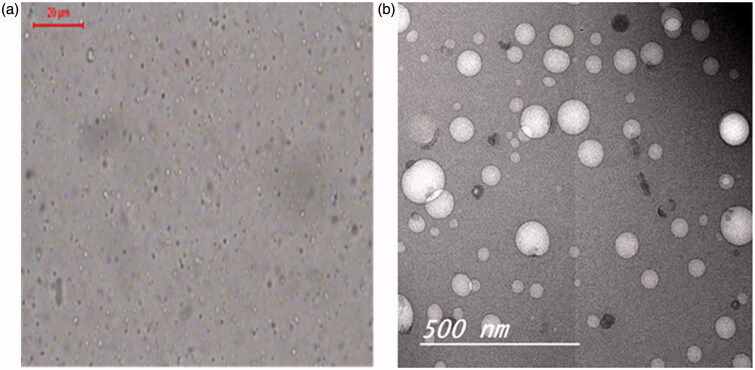
Photomicrograph (a) and TEM micrograph (b) of the optimized bosentan RCRPC.

**Figure 2.**
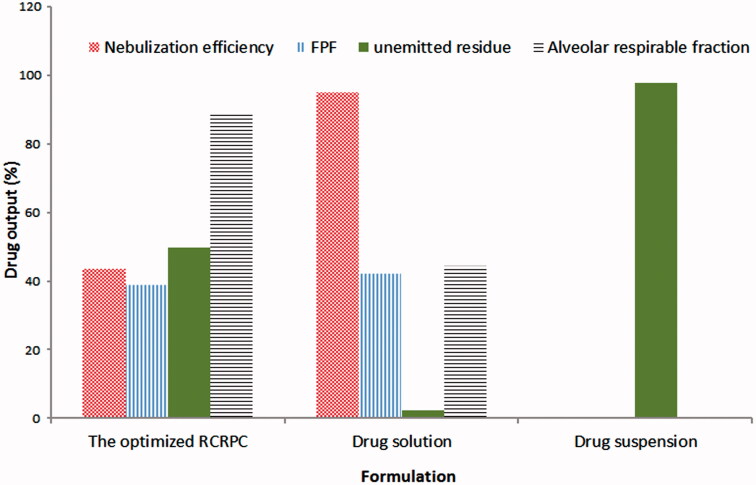
Aerodynamic parameters of the optimized bosentan RCRPC, drug solution and drug suspension after nebulization in a TSI.

Figure 1(a,b) shows the morphological examination of the optimized bosentan-loaded RCRPC using photomicroscope and TEM. The photomicrographs showed that all the prepared nanoparticles were discrete, non-agglomerated and spherical in shape. TEM micrograph (Figure 2b) demonstrates the morphology of the optimized formulation. As obvious, the polymeric nanoparticles are spherical with size ranging between (100 and 350) nm which has been reported to be appropriate for intratracheal administration (Hara et al., 2008).

### Physical characterization of the lyophilized optimized RCRPC

#### DSC

The DSC thermograms of bosentan, PLGA, mannitol, PM of (PLGA, bosentan and mannitol) and the lyophilized optimized RCRPC were performed. Bosentan monohydrate exhibits a broad endothermic peak at about 108 °C. PLGA thermogram showed endothermic peak at 29.72 °C. The thermogram of mannitol showed a sharp endotherm at 172.59 °C corresponding to its melting point. The PM showed endothermic peaks at 29.77 °C, 116.97 °C and 172.65 °C respectively indicating the presence of the drug in the crystalline form. The thermogram of the lyophilized optimized RCRPC was characterized by disappearance of the endothermic peak of the drug indicating that the drug is dispersed in PLGA matrix in an amorphous form. Moreover, a new endothermic peak at 154.93 °C appeared which is probably related to the mannitol tendency to crystalize (Yu et al., 1998; Chacon et al., 1999).

#### X-ray diffraction (XRD)

The XRD diffractograms of bosentan, PLGA, mannitol, PM of (PLGA, bosentan and mannitol) and the lyophilized optimized RCRPC were performed. The diffractogram of bosentan revealed its crystalline nature as indicated by its prominent diffraction peaks with the highest intensity at 2*θ* of 9.6, 16.1, 17.1, 18.4 and 21.8 (Rao et al., 2013). The diffractograms of PLGA and mannitol indicated the amorphous and highly crystalline nature, respectively, of these materials. The lyophilized optimized RCRPC showed a typical diffuse pattern with complete absence of the numerous distinctive peaks of bosentan indicating that drug was molecularly dispersed in PLGA matrix.

### *In vitro* aerosol delivery of the optimized bosentan-loaded RCRPC in a TSI

The aim of this study was to investigate the aerodynamic properties of RCRPR dispersion compared to drug solution and drug suspension. Figure 2 shows the aerodynamic parameters of the optimized RCRPC, drug solution and drug suspension after nebulization in a TSI. Drug solution gave the highest NE (95.17 ± 2.9%) followed by the optimized RCRPC (43.75 ± 1.2%) and finally drug suspension (0%). After nebulization process, drug suspension showed no drug nebulization and approximately the total dose was detected in the nebulizer cup (97.61 ± 1.8%). There was an inverse relationship between the droplet size and the NE. Upon nebulization, liquid is sheared by compressed air to be converted into small droplets which escape outside the nebulizer cup and large droplets which are baffled, returned and remained in the nebulizer cup. This process is very rapid in case of drug solutions which allow nebulization of the whole liquid especially solutions containing surfactants which are capable to reduce the surface tension of the liquid and the droplet size. However, in case of colloidal dispersion such as RCRPC, the nebulization process takes more time to separate the aggregation between nanoparticles and the adjacent liquid sheath. During this process, evaporation occurred and an increase in the concentration in the nebulizer cup took place. Increasing the cup concentration would consequently increase the aerodynamic diameter and hence decreased the drug NE percentage (Gonda, 1985; Abd-Elbary et al., 2008).

Interestingly, although the NE of drug solution was significantly higher than that of the optimized RCRPC, the amount of drug deposited in the lower stage of the impinger (i.e. FPF) of both formulations was very close (42.41 ± 1.6% and 39.06 ± 2.4%, respectively). The upper impingement chamber is designed such that at a flow rate of 60 L/min through the impinger, the particle cutoff size is 6.4 μm. Generally, particles smaller than 6.4 μm pass into the lower impingement chamber (British Pharmacopia, 2014). This observation confirmed that the nebulized droplets of RCRPC had lower droplet size than that of drug solution. The high ARF, 89.28 ± 3.1%, of the optimized RCRPC indicates that approximately 90% of the nebulized dose reaches the lower chamber of the impinger due to the small diameter of the nebulized droplets. However, ARF of the drug solution was 44.56 ± 1.9%. In other words, the fraction of small droplets (<6.4 mm) in the nebulized RCRPC were about two folds higher than that of the nebulized drug solution. It has been previously stated in literature that the nebulization performance is highly affected by formulation physicochemical properties (Nasr et al., 2012). The dispersed nanospheres (<500 nm) of RCRPC could act as nuclei on which layers of water are deposited forming the nebulized liquid droplet that are most desirable for alveolar deposition. These findings indicate that colloidal dispersions need more time to be nebulized and once nebulized; they are incorporated into “respirable nebulized droplets” appropriate for deep lung deposition (distal or peripheral).

### *In vivo* pulmonary absorption of bosentan-loaded RCRPC

Pharmacokinetic studies following pulmonary delivery of nanocarrier systems have been frequently performed in rats, as blood samples at all sampling times can be collected in one rat. Moreover, although major differences in lung anatomy exist between humans and rodents, these differences do not have a significant impact on the optimal size of aerosol particles for alveolar deposition. The optimum particle size required for alveolar deposition in human and rats are 1–5 μm and 3.5 μm, respectively (Fernandes & Vanbever, 2009). Sample size (*n* = 8 for each group) was selected not based on statistical consideration but rather on literature review (Yuan et al., 2015; Varshosaz et al., 2016; Zhang & Xu, 2016).

**Figure 3.**
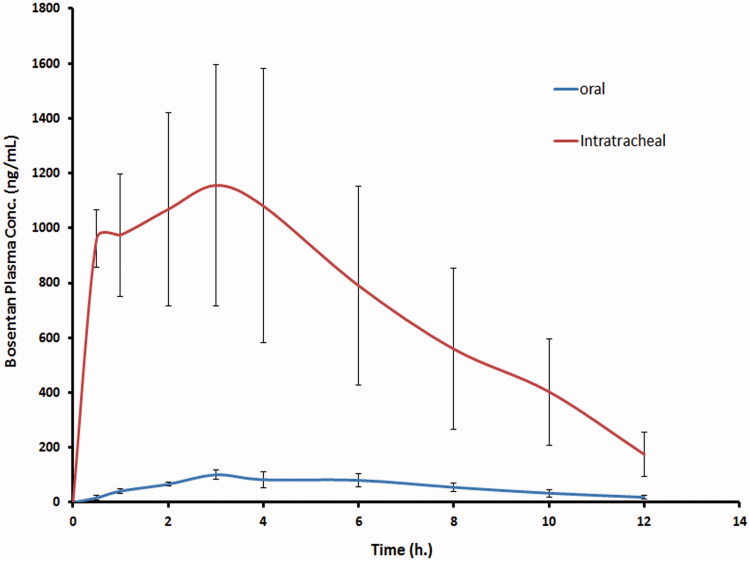
The mean bosentan concentrations in plasma of rats after intratracheal delivery of the optimized bosentan RCRPC compared to the oral administration of bosentan suspension.

As shown in Table 2 and Figure 3, the mean *C*_max_ estimated in plasma after oral administration of drug suspension and intratracheal delivery of the optimized RCRPC were 105.1 ± 18.12 ng/mL and, 1264.8 ± 323.68 ng/mL, reached after times (*T*_max_) 4.0 ± 1.41 h and 2.62 ± 1.49 h, respectively. The differences between the two formulations for *C*_max_ was found to be statistically significantly different (*p* = 0.0003). The non-parametric test (Kruskal–Wallis test) showed a non-significant difference between *t*_max_ of both treatments. The mean AUC_0–_*_t_* estimated from the intratracheal RCRPC (8649.8 ± 3319.7 ng h/mL), which reflects the total amount of drug absorbed over the 12 h time period, was determined to be about 12.71 folds higher and statistically significantly different (*p* = 0.0030) compared to the mean AUC_0–_*_t_* estimated from the oral drug suspension (680.2 ± 133.8 ng h/mL). The improved bioavailability of bosentan from the intratracheal RCRPC could be explained based on the capability of bosentan nanoparticles to adhere to the bronchial and lung tissue and sustain the release of drug at the adsorption site. Moreover, the intratracheal delivery of bosentan avoids the hepatic first-pass effect. It was reported that PLGA nanospheres were retained in type 1 alveolar epithelium cell, alveolar space and blood basement membrane after intratracheal drug administration then absorbed by transcellular endocytosis through type 1 alveolar epithelium cell (Hara et al., 2008).

**Table 2.** The mean pharmacokinetic parameters of bosentan in plasma after intratracheal delivery of the optimized RCRPC and oral administration of bosentan suspension to rats.

Parameter	Oral drug suspension	Intratracheal RCRPC	Significance (*p* value)
*C*_max_ (ng/mL)	105.1 ± 18.12	1264.8 ± 323.68	0.0003
*T*_max_ (h)	4.0 ± 1.41	2.62 ± 1.49	0.2296
AUC_0–_*_t_* (ng.h/mL)	680.2 ± 133.8	8649.8 ± 3319.7	0.0030
AUC_0–∞_ (ng.h/mL)	760.25 ± 167.66	9592.61 ± 3551.65	0.0025
*T*_1/2_ (h)	2.8 ± 0.59	3.17 ± 0.47	0.3723

Values are expressed as mean ± SD.

### Histopathological examination of rat lung tissue

**Figure 4.**
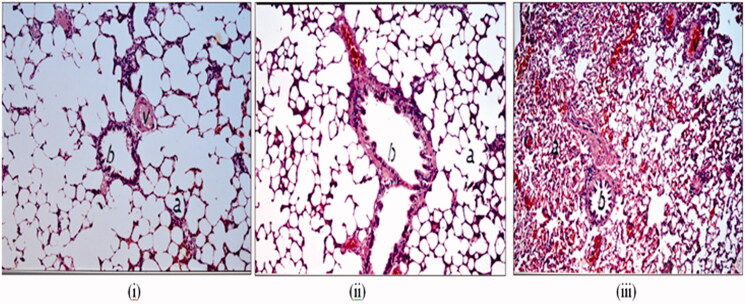
Histological microscopic features of rat lung tissue after intratracheal administration of PBS as a negative control (i), oral administration of oral bosentan suspension (ii) and intratracheal administration of bosentan RCRPC (iii).

The stained rat lung tissues showed no histopathological changes after 12 h of administration of oral drug suspension compared to the PBS negative control administered intratracheally as evident in Figure 4. There was normal histological structure of the bronchioles, air alveoli and blood vessel with alveolar walls being intact and displaying normal morphology in both groups (Figure 4i,ii). The congestive effect of oral bosentan on the pulmonary blood vessels disappeared after 12 h of oral administration of drug suspension which necessitates higher frequency of administration (twice daily). This observation correlates well with the pharmacokinetic study where very low plasma bosentan concentration (<20 ng/mL) was detected. However, Figure 4(iii) shows histological features of rat lung tissues after intratracheal administration of bosentan RCRPC where congestion in the interalveolar and perialveolar blood vessels and capillaries was observed. The lack of emphysema and the significant congestion of alveolar blood vessels confirmed the vasodilation effect of bosentan on the pulmonary arteries as endothelin antagonist. The congestive effect of intratracheal bosentan RCRPC on the pulmonary blood vessels was prominent even after 12 h of administration which correlates well with the pharmacokinetic study where high bosentan concentration (>170 ng/mL) was detected in the plasma of rats which was higher than that of *C*_max_ of orally administered bosentan suspension (105.1 ± 18.12 ng/mL). The prolonged congestive effect of intratracheal bosentan RCRPC further confirms the retention of drug-loaded PLGA nanoparticles in deep lung tissues allowing slow release of bosentan. Hara et al. had reported that the possibility to induce tissue damage caused by the excessive immune response from the deposition of PLGA nanospheres in the lung tissues was very low, because these nanospheres were not treated as foreign substances (Hara et al., 2008).

## Conclusion

In this study, RCRPC, bosentan-loaded PLGA colloidal dispersion, was successfully prepared in nano-size range (<500 nm). *In vitro* nebulization showed that RCRPC could be incorporated into “respirable nebulized droplets” appropriate for deep lung deposition better than drug solution. Pulmonary delivery of bosentan-loaded RCRPC showed improved bioavailability (12.71 folds) and sustained vasodilatation effect on the pulmonary blood vessels (more than 12 h) compared to the oral bosentan suspension. The adverse effects of ERA could be reduced by inhalation of RCRPC loaded with much lower doses than the oral one. Bosentan-loaded RCRPC administered via the pulmonary route may therefore constitute an advance in the management of PAH. Further studies for pharmacokinetic and tolerability studies of bosentan-loaded nanocarrier systems in human are presently investigated.
